# Expression of H_2_S in Gestational Diabetes Mellitus and Correlation Analysis with Inflammatory Markers IL-6 and TNF-*α*

**DOI:** 10.1155/2020/3085840

**Published:** 2020-03-23

**Authors:** Yucui Teng, Shuxia Xuan, Ming Jiang, Li Tian, Jinjing Tian, Qian Chang

**Affiliations:** ^1^Department of Obstetrics, The Second People's Hospital of Liaocheng, Linqing, Shandong Province, China 252600; ^2^Department of Laboratory, The Second People's Hospital of Liaocheng, Linqing, Shandong Province, China 252600

## Abstract

**Background:**

Gestational diabetes mellitus (GDM) is a severe threat to the health of both mother and child. The pathogenesis of GDM remains unclear, although much research has found that the levels of hydrogen sulfide (H_2_S) play an important role in complications of pregnancy.

**Methods:**

We collected venous blood samples from parturient women and umbilical vein blood (UVB) and peripheral venous blood (PVB) samples one hour after childbirth in the control, GDM-, and GDM+ groups in order to determine the concentration of glucose and H_2_S in plasma; to measure levels of TNF-*α*, IL-1*β*, IL-6, TGF-*β*1, and ADP in parturient women and the UVB of newborns; and to find the correlation of H_2_S with regression.

**Results:**

We found that, with the elevation of glucose, the level of H_2_S was decreased in GDM pregnant women and newborns and the concentrations of IL-6 and TNF-*α* were upregulated. With regression, IL-6 and TNF-*α* concentrations were positively correlated with the level of blood glucose and negatively correlated with H_2_S concentration.

**Conclusion:**

This study shows that downregulation of H_2_S participates in the pathogenesis of GDM and is of great significance in understanding the difference of H_2_S between normal and GDM pregnant women and newborns. This study suggests that IL-6 and TNF-*α* are correlated with gestational diabetes mellitus. The current study expands the knowledge base regarding H_2_S and provides new avenues for exploring further the pathogenesis of GDM.

## 1. Introduction

Gestational diabetes mellitus (GDM) is a type of glucose intolerance first discovered during pregnancy [1] and is one of the most common complications of pregnancy, resulting in hyperglycemia of variable severity. It is a severe threat to the health of both mother and child. The pathogenesis of GDM remains unclear, but the discovery of gas-signaling molecules provides a new facet for GDM research. Hydrogen sulfide (H_2_S) is the third endogenous gas-signaling molecule discovered in recent years and is produced mainly by cysteine under the action of CSE (cystathionine-*γ*-lyase) in cardiovascular tissue [2, 3]. Previous research has found that H_2_S levels were downregulated in gestational hypertension (GH) patients [4, 5]. This suggested that H_2_S plays an important role in complications of pregnancy as well, making understanding of the levels and mechanisms of H_2_S in GDM an important research topic. Here, we study the different concentrations of plasma H_2_S in patients and the related mechanism to investigate the role of H_2_S in the pathogenesis of GDM.

## 2. Materials and Methods

### 2.1. Diagnosis of GDM

Diagnoses of GDM were made following the recommended diagnostic criteria of the International Association of the Diabetes and Pregnancy Study Groups 2010 (IADPSG 2010). These new criteria recommend a universal 75 g oral glucose tolerance test (OGTT) screening for pregnant women at 24-28 weeks, which employs more rigorous glucose level cut-offs. The diagnostic thresholds for OGTT include a fasting blood sugar (FBS) value of 5.1 mmol/L (92 mg/dL) and one-hour and two-hour posttolerance test blood sugar values of 10.0 and 8.5 mmol/L (180 and 153 mg/dL), respectively. GDM was diagnosed if any of the blood sugar values reached or exceeded these thresholds.

### 2.2. Study Subjects and Groups

*Inclusion and exclusion criteria*: pregnant women and newborns meeting the following criteria were included in our study: single pregnancy; full-term pregnancy, primipara/second-child pregnant women without abortion or induction of labor, and no diabetes mellitus or GDM prior to the current pregnancy.

Samples for the current study were collected from 362 patients who underwent routine pregnancy check-ups and delivery in the Department of Obstetrics at the Second People' s Hospital of Liaocheng, Shandong, China, between January 2015 and December 2018. Signed informed consent forms were obtained from all patients prior to their inclusion in this study, and our experimental protocols were approved by the ethics committee of the Second People's Hospital of Liaocheng. Patient charts were reviewed to obtain the venous blood of the parturient 5 days before childbirth and umbilical vein blood (UVB) and peripheral venous blood (PVB) one hour after delivery of newborns.

The patients (parturient and newborns) were divided into three groups: the control group (ctrl) was composed of normal pregnant women without complications during pregnancy, especially the 75 g OGTT at 24-28 weeks; the GDM group (GDM-) was composed of pregnant women diagnosed with GDM at 24-28 weeks and well controlled with a diet program change and/or medical treatment (such as insulin injection or MTF (metformin) oral intake); and the hyperglycemia GDM group (GDM+) included pregnant women diagnosed with GDM at 24-28 weeks and whose hyperglycemia was poorly controlled, regardless of treatment.

### 2.3. Sample Preparation and Preservation

Venous blood samples from parturient women and the UVB of newborns were collected from each group and placed in dry tubes treated with heparin anticoagulant. The plasma was centrifuged at 800 rpm at 4°C, separated, and preserved at -70°C.

The blood glucose levels in the one-hour PVB of newborns were measured with a fast glucose meter (OMRON, HGM-123, OMRON Corporation, Suzhou, Jiangsu, China) and recorded.

The blood glucose, insulin, bilirubin, hemoglobin, calcium, phosphorus, and magnesium of UVB were measured by electrochemical methods using an automatic biochemical instrument (Siemens, ADVIA® XPT, Siemens Healthineers, Shanghai, China).

Insulin resistance (IR) was calculated according to the equation IR = FBS × FIRI/22.5 (FIRI: fasting insulin).

It was to be noted that the patient's information was just provided with the patient's identification number as the determination of H_2_S in plasma and ELISA, so the authors involved in data measurements were completely blind to which group the blood samples belonged.

### 2.4. Determination of H_2_S in Plasma

Plasma H_2_S concentrations were measured using the sulfide-sensitive electrode method (PXS-270, Shanghai, China) as described by Sun et al. [6].

A total of 0.1 mL plasma was added to a tube containing 0.5 mL 1% zinc acetate and 2.5 mL distilled water. Into this tube, 0.5 mL of 20 mmol/L N,N-dimethyl-p-phenylenediamine dihydrochloride in 7.2 mmol/L HCl and 0.4 mL of 30 mmol/L FeCl_3_ in 1.2 mmol/L HCl were added and incubated for 20 min at room temperature (RT). The plasma protein was removed by the addition of 1 mL of 10% trichloroacetic acid to the reaction mixture and centrifugation at 3000 r/min for 15 min. The optical absorbance of the resulting solution was measured at 670 nm using a spectrometer (Shimadzu UV 2100; Shimadzu, Kyoto, Japan).

All samples were assayed in duplicate, and the concentration in the solution was calculated against a calibration curve of NaHS (3.125-250 mmol/L).

### 2.5. ELISA

Double-antibody human TNF-*α*, IL-1*β*, IL-6, and TGF-*β*1 ELISA kits were purchased from the Beijing BLKW Biotechnology Co., Ltd. (Beijing, China). Human Adiponutrin ELISA kits (Catalog #: EL-PRELIM) were bought from RayBiotech Life (Peachtree Corners, Georgia, USA).

All samples and standards of different concentrations were assayed according to the manufacturers' instructions. Briefly, samples (100 *μ*L/well) were added and sealed and incubated at RT for 120 minutes. The plate was washed five times and patted dry. Biotinylated antibody working solution was added (100 *μ*L/well) and the plate sealed and incubated at RT for 60 minutes. The plate was washed again 5x and patted dry. Enzyme conjugate working fluid (100 *μ*L/well) was added and the plate sealed and incubated at RT for 20 minutes. The plate was washed and dried a final time, and the color-developing substrate solution was added (TMB 100 *μ*L/well) and incubated at RT for 20 minutes. 50 *μ*L/well stop solution was added, and the OD_450_ was measured immediately after mixing.

### 2.6. Statistical Analysis

Statistical analyses were performed using SPSS 17.0 (SPSS Inc., Chicago, IL, USA), and results were expressed as mean ± standard deviation (SD). The differences among independent samples were tested by one-way ANOVA. The relation between two groups was analyzed by linear logistic regression. We performed independent sample *t*-tests, and *p* < 0.05 was considered statistically significant.

## 3. Results

### 3.1. The Difference of Clinicopathological Characteristics

According to our inclusion and exclusion criteria, excluding samples without complete information, 185 women and newborns in the control group, 125 cases in the GDM- group, and 52 cases in the GDM+ group were involved in our analysis.

We analyzed the age, ratio of first/second child, weight, BMI (body mass index, the weight in kilograms divided by the square of the height in meters, kg/m^2^) of parturient, and the gender and weight of newborns. As shown in our results, there were no differences in the age of expectant mothers (*p* = 0.397), and no significant differences in the ratio of parity (*p* = 0.952), height (*p* = 0.944), or weight of either pregnant women or newborns (*p* = 0.176, 0.138), or gender of offspring (*p* = 0.749) were observed. The BMI of the 3 groups also showed no differences (*p* = 0.249).

### 3.2. H_2_S Was Downregulated in GDM

**Figure 1:**
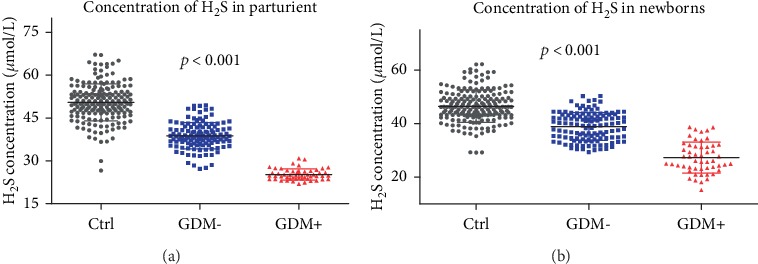
Concentration of H_2_S detected in the venous blood of parturient women and the UVB of newborns. (a) Concentration of H_2_S detected in the venous blood of pregnant women was decreased among the control, GDM-, and GDM+ groups. (b) Concentration of H_2_S detected in the UVB of newborns was decreased among the control, GDM-, and GDM+ groups.

The concentration of H_2_S from pregnant women and the UVB of newborns was measured using the sulfide-sensitive electrode method. The average concentration was downregulated in the well-controlled GDM- patients and obviously decreased in poorly controlled GDM+ patients. The average concentrations were 51.76 ± 4.98, 38.78 ± 4.57, and 25.22 ± 1.94 *μ*mol/L in the control, GDM-, and GDM+ groups, respectively, in women (Figure 1(a), *p* < 0.001) and 48.21 ± 4.55, 38.90 ± 5.16, and 27.29 ± 5.80 *μ*mol/L, respectively, in newborns (Figure 1(b), *p* < 0.001).

### 3.3. Hyperglycemia in Pregnancy Leads to Hypoglycemia in Newborns

**Figure 2:**
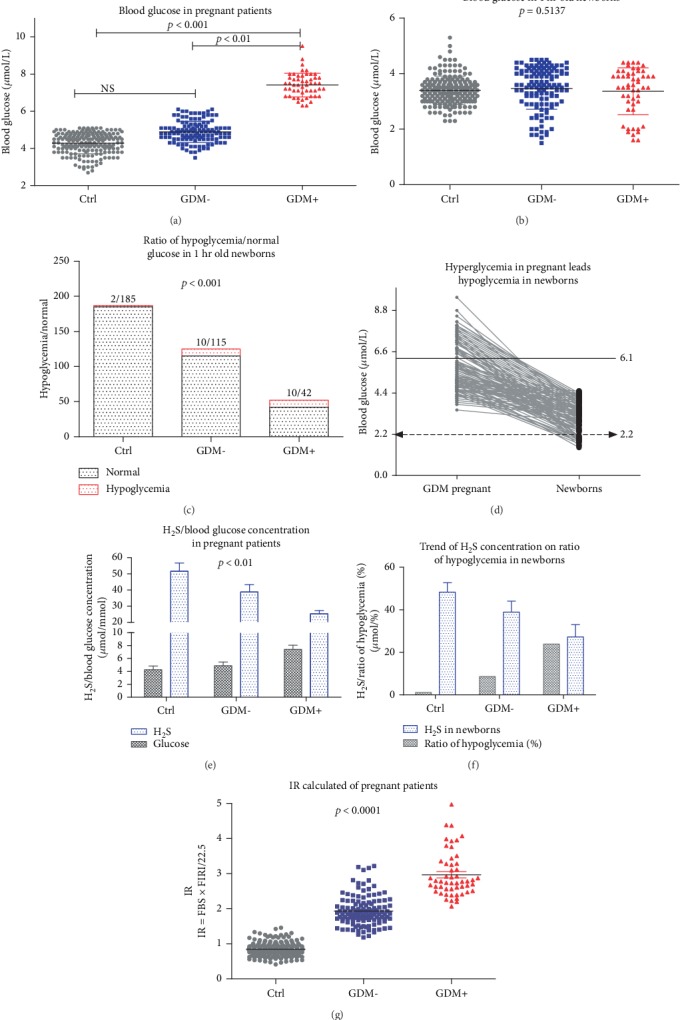
Blood glucose level and its relation with H_2_S concentration and hypoglycemia in newborns. (a) Blood glucose was elevated among pregnant women in the control, GDM-, and GDM+ groups. (b) The 1-hour blood glucose in newborns showed no differences among the control, GDM-, and GDM+ groups. (c) The ratio of hypoglycemia in newborns, 2/185, 10/115, and 10/42, showed great differences. (d) Comparison of blood glucose concentrations in pregnant women and 1-hour-old newborns. With the increase of blood glucose level, the 1-hour blood glucose of newborns was downregulated, and elevated blood glucose seems to be a leading factor for hypoglycemia. (e) Among these 3 groups, the elevated glucose in pregnant women was related to the decreased expression of H_2_S. (f) In newborns, the decreased H_2_S concentration seems an inducement of the ratio of hypoglycemia in newborns. (g) With the increase of hyperglycemia, the level of IR was upregulated in GDM-/+ pregnant women.

As shown in Figure 2(a), the average blood glucose levels were no different between the control group and the GDM- group, but the GDM+ group was different from those two groups (Figure 2(a), *p* < 0.01 and *p* < 0.001, respectively). The one-hour glucose of newborns showed an opposite tendency from expectant women. The average glucose showed no difference (Figure 2(b), *p* = 0.514), but the ratio of hypoglycemia in newborns, 2/185, 10/115, and 10/42, showed great differences (Figure 2(c), *p* < 0.001). Compared to the concentration of blood glucose in parturient and 1-hour newborns, with the increase of blood glucose levels, the 1-hour blood glucose of newborns was reduced, and the elevated blood glucose seemed to be a leading factor of hypoglycemia (Figure 2(d)). This result shows that hyperglycemia in pregnant women leads to hypoglycemia in 1-hour newborns. Among these 3 groups, the elevated glucose in pregnancy was related to the decreased expression of H_2_S (Figure 2(e), *p* < 0.01). In newborns, the reduced expression of H_2_S seems to be an inducement of the ratio of hypoglycemia in newborns (Figure 2(f)).

Biochemical markers, such as incidence of physiological/pathological jaundice (97/185, 73/125, and 31/52), hemoglobin of UVB (178.26 ± 6.27, 174.56 ± 4.18, and 177.34 ± 6.19, *p* = 0.0611) was measured; the levels showed no significant differences among the 3 groups. Similar results were observed in calcium, phosphorus, and magnesium levels in UVB (data was not shown).

With the increase of hyperglycemia, the level of calculated IR was increased in GDM-/+ pregnancy (Figure 2(f), *p* < 0.0001).

### 3.4. Glucose Levels Influence Inflammatory Cytokines IL-6, TNF-*α*, and ADP in Pregnant Women and Newborns

**Figure 3:**
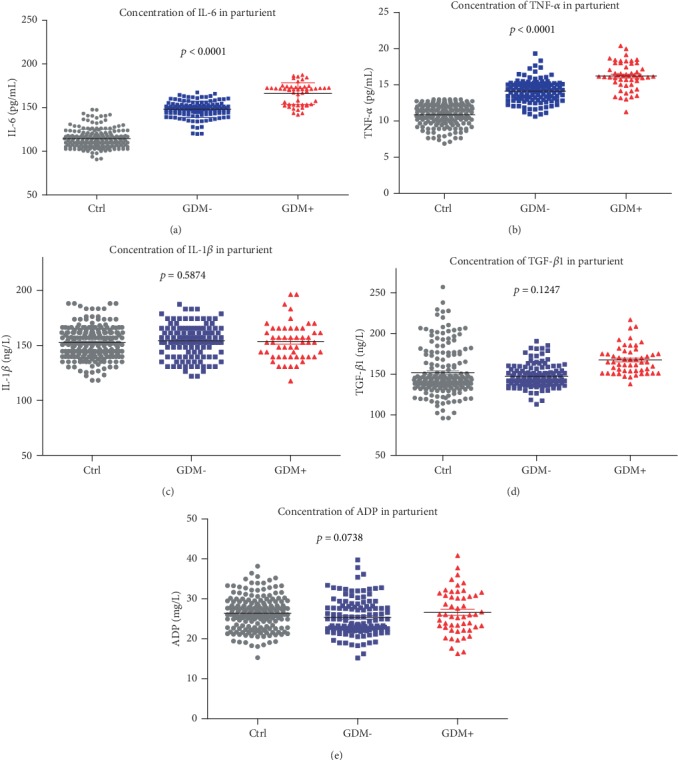
The level of glucose influenced the inflammatory cytokines TNF-*α*, IL-1*β*, IL-6, TGF-*β*1, and ADP in pregnant women. (a, b) The expression of IL-6/TNF-*α* was increased among the control, GDM-, and GDM+ groups in pregnant women. (c–e) The expression of IL-1*β*, TGF-*β*1, and ADP showed no differences among the control, GDM-, and GDM+ groups in pregnant women.

To understand better the mechanism of glucose in GDM patients, we measured TNF-*α*, IL-1*β*, IL-6, TGF-*β*1, and ADP in our study. As shown in Figures 3(a)–3(c), as the glucose concentration increased in pregnant women among the three groups, the levels of these 5 cytokines increased as well,, but only IL-6 (Figure 3(a), *p* < 0.0001) and TNF-*α* (Figure 3(b), *p* < 0.0001) showed statistically significant increases in both groups. IL-1*β* (Figure 3(c), *p* = 0.5874), TGF-*β*1 (Figure 3(d), *p* = 0.1247) and ADP (Figure 3(e), *p* = 0.0738) showed no significant difference.

**Figure 4:**
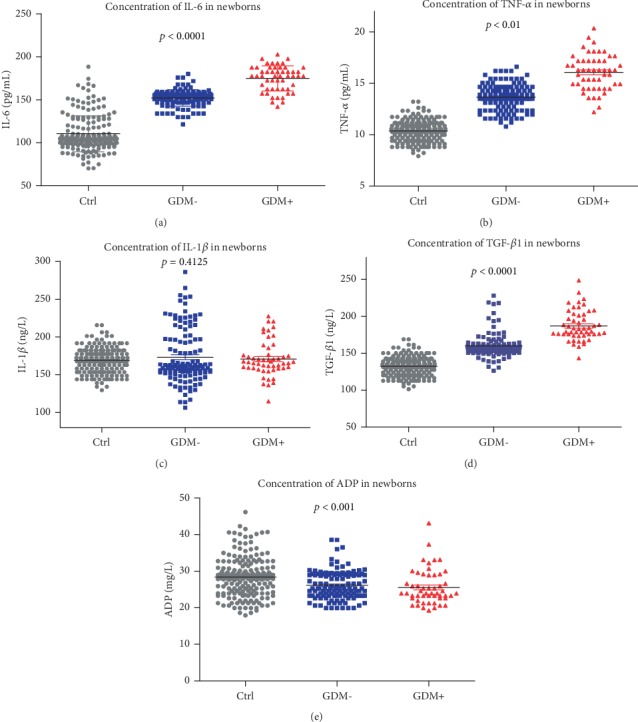
The level of glucose influenced the inflammatory cytokines TNF-*α*, IL-1*β*, IL-6, TGF-*β*1, and ADP in newborns. (a) The expression of IL-6/TNF-*α* was increased among the control, GDM-, and GDM+ groups. (c) The expression of IL-1*β* showed no differences among the control, GDM-, and GDM+ groups. (d) The expression of TGF-*β*1 was increased among the control, GDM-, and GDM+ groups in newborns. (e) The expression of ADP was decreased among the control, GDM-, and GDM+ groups.

In newborns, the results showed that IL-6 (Figure 4(a), *p* < 0.0001), TNF-*α* (Figure 4(b), *p* < 0.01), TGF-*β*1 (Figure 4(d), *p* < 0.0001), and ADP (Figure 4(e), *p* < 0.001) displayed significant differences between the groups, but IL-1*β* (Figure 4(d), *p* = 0.4125) resulted in no significant changes.

### 3.5. H_2_S Concentration Influences Glucose and IL-6 Levels in GDM Patients

**Figure 5:**
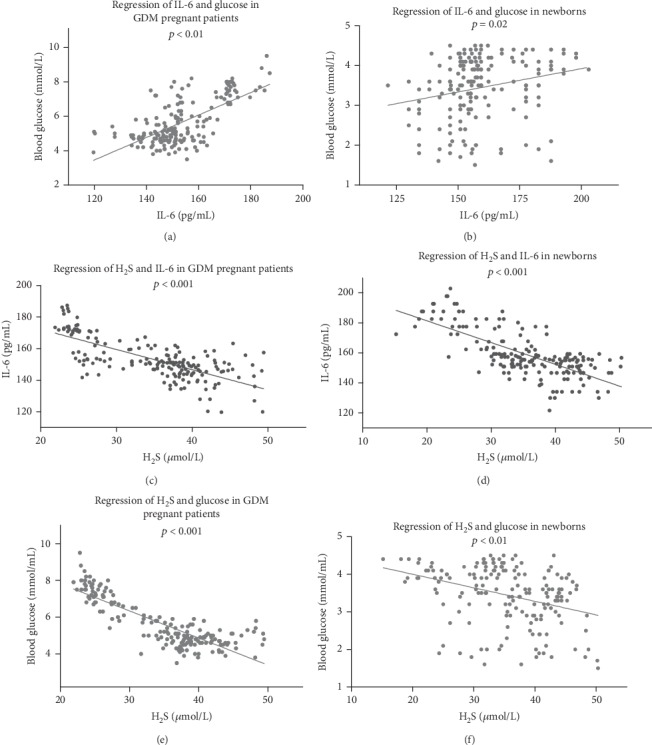
The concentration of H_2_S influenced the glucose and IL-6 levels in GDM patients. (a) The levels of IL-6 and blood glucose were positively correlated. (c, e) The concentration of H_2_S was negatively correlated with levels of IL-6 and glucose. (b) IL-6 and glucose were positively correlated in the UVB of newborns. (d, f) H_2_S was negatively correlated with IL-6 and glucose in newborn UVB.

In order to elucidate the function of H_2_S in GDM patients, both with well-controlled and poorly controlled blood glucose levels, we performed regression analysis on the relationship between H_2_S, IL-6, and blood glucose. As shown in Figure 5, the level of IL-6 and glucose showed a positive correlation (Figure 5(a), *r*^2^ = 0.4358, *p* < 0.01), but the concentration of H_2_S was negatively correlated with levels of IL-6 and glucose (Figure 5(c), *r*^2^ = 0.521, *p* < 0.001, and Figure 5(e), *r*^2^ = 0.706, *p* < 0.001).

In newborn UVB samples, the correlation of H_2_S with IL-6 and blood glucose followed the same pattern as in GDM patients. IL-6 and glucose were positively correlated (Figure 5(b), *r*^2^ = 0.5342, *p* = 0.02), but H_2_S was negatively correlated with IL-6 and glucose (Figure 5(d), *r*^2^ = 0.509, *p* < 0.001, and Figure 5(f), *r*^2^ = 0.1217, *p* < 0.001).

### 3.6. H_2_S Concentration Influences the Levels of TNF-*α* in GDM Patients

**Figure 6:**
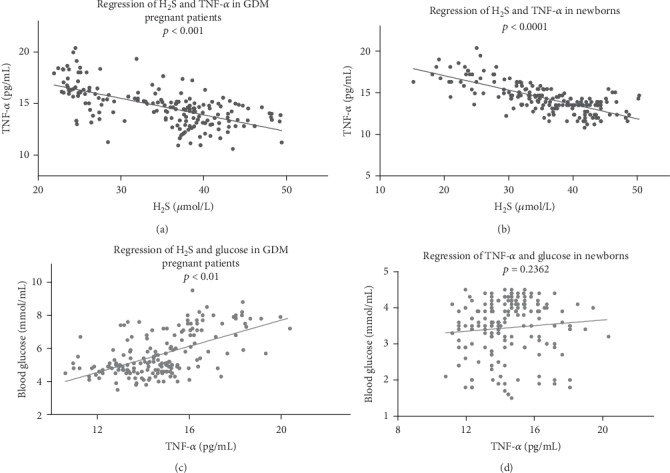
H_2_S concentration influenced the level of TNF-*α* in GDM patients. (a, b) H_2_S concentration was negatively correlated with TNF-*α* levels in pregnant women and newborns. (c) The levels of TNF-*α* and glucose showed a positive correlation in pregnant women. (d) The levels of TNF-*α* and glucose showed no significant correlation in newborns.

As with IL-6, we analyzed the relationship of H_2_S with TNF-*α* and blood glucose. As shown in Figure 6, the concentration of H_2_S was negatively correlated with levels of TNF-*α* in pregnant women and newborns (Figure 6(a), *r*^2^ = 0.3912, *p* < 0.001, and Figure 6(b), *r*^2^ = 0.5278, *p* < 0.001). The levels of TNF-*α* and glucose were positively correlated in pregnant women (Figure 6(c), *r*^2^ = 0.3312, *p* < 0.001) but showed no significance in newborns (Figure 6(d), *r*^2^ = 0.0008, *p* = 0.2362).

### 3.7. H_2_S Concentration Influences the Levels of TGF-*β*1 and ADP in GDM Patients

We analyzed the relationship of H_2_S with ADP and blood glucose. As shown in [Supplementary-material supplementary-material-1], the concentration of H_2_S was negatively correlated with levels of TGF-*β*1 and ADP in pregnant women and newborns ([Supplementary-material supplementary-material-1], *r*^2^ = 0.1613 and 0.1692, *p* < 0.0001 and [Supplementary-material supplementary-material-1], *r*^2^ = 0.4482 and 0.5245, *p* < 0.001).

The levels of TGF-*β*1 and glucose were positively correlated in pregnant women ([Supplementary-material supplementary-material-1], *p* < 0.0001) but showed no significance in newborns ([Supplementary-material supplementary-material-1], *p* = 0.6876); as per analysis shown in Figures 3(d) and 4(d), the results showed no significance.

The levels of ADP and glucose showed the same results, and they were positively correlated in pregnant women ([Supplementary-material supplementary-material-1], *p* < 0.0001) but showed no significance in newborns ([Supplementary-material supplementary-material-1], *p* = 0.1249); as per analysis shown in Figures 3(e) and 4(e), the results showed no significance.

## 4. Discussion

GDM is one of the common perinatal complications, and its pathogenesis remains not yet fully understood. Many studies have shown that the risk of type 2 diabetes mellitus (T2DM) in pregnant women with GDM has increased significantly [7]. GDM is considered an early stage of T2DM. The classic view held that the antagonistic effects of prolactin, prolactin, glucocorticoid, and progesterone on insulin secretion and insulin resistance during pregnancy were the main causes of GDM. Although there exists insulin resistance in every pregnant woman, only 4% of pregnant women will eventually develop GDM, which suggests that there may be other reasons for the pathogenesis of GDM [8]. In addition, studies have found that the occurrence of GDM is related not only to insulin secretion and dysfunction but also to some inflammatory and fatty factors [9, 10]. However, the etiology of GDM is still not fully understood.

Recently, H_2_S has been found to have important biological functions. As an endogenous gas-signaling molecule, H_2_S has many biologically important characteristics, such as small molecular weight, continuous production, rapid diffusion, and wide effects. It is generated in cardiovascular tissues under the action of CSE with cysteine as the main substrate [2, 3]. Some studies have found that the disorder of the H_2_S/CSE system is important in the pathogenesis of many vascular injury diseases [11, 12]. It was also found that H_2_S inhibits insulin release and regulates beta-cell survival [13]. Therefore, H_2_S may play a role in the pathogenesis of diabetes mellitus. The purpose of this study was to investigate whether there were differences in H_2_S in the venous blood of GDM patients and normal pregnant women and the UVB of newborns and to explore the mechanism of these differences in the pathogenesis of GDM.

This study found that plasma levels of H_2_S in the venous blood and neonatal umbilical vein blood of GDM patients were significantly lower than those of normal pregnant women, suggesting that H_2_S is involved in the pathogenesis of GDM. As the average blood glucose rose among the 3 groups, the H_2_S concentrations decreased. The state of hyperglycemia in GDM patients was closely related to the decrease of H_2_S concentration.

Previous studies have found that, in high glucose-induced insulin resistance rat model animals, plasma H_2_S levels and the expression of CSE were significantly lower than those in the control group. The pathogenesis of GDM was closely related to insulin resistance, so we speculated that the pathogenesis of GDM was closely related to the downregulation of the H_2_S/CSE system. The results of this study validate this hypothesis. In the cardiovascular system and in the pancreas, CSE is a rate-limiting enzyme catalyzing H_2_S production. Therefore, in the venous blood of GDM patients, whether the decrease of H_2_S concentration is related to the decrease of CSE activity or expression remains to be studied further.

In the current study, the blood glucose levels of the GDM- group were maintained in the normal range by dietary control or insulin treatment, and hypoglycemia still occurred in newborns. The ratio of hypoglycemia in newborns of the GDM- group was significantly greater than that of the normal control group 1 hour after birth. The incidence of hypoglycemia in newborns of the GDM+ group was higher, and the average blood glucose value was lower, which suggested that hyperglycemia in GDM patients is an important risk factor for neonatal hypoglycemia.

As measured in our study, the biochemical markers bilirubin, hemoglobin, calcium, phosphorus, and magnesium showed no significant differences in the UVB among the 3 groups. For GDM newborns, however, they are important risk factors to monitor to ensure the health and safety of newborns.

Many inflammatory factors, such as TNF-*α* and IL-6, have strong biological activities and act in the inflammatory response of many diseases. IL-6 has been proven to be involved in many inflammatory reactions and has the ability to regulate the balance of energy metabolism [14]. It has been reported that the serum levels of IL-6 and hs-CRP (high-sensitivity C-reactive protein) in patients with GDM are significantly increased [10], but the mechanism is not fully understood, and the causal relationship with insulin resistance is still controversial. It has been reported that the initiating factor of insulin resistance may be chronic inflammation, and it has been speculated that IL-6 may be involved in the occurrence of GDM [15, 16].

Some studies suggest that TNF-*α* and IL-6 interact to interfere with insulin signal transduction, which leads to insulin resistance and eventually to the occurrence of GDM [17, 18]. IL-6 may be involved in the immune response to inflammation and play a bridge role between local tissue damage and systemic immune response. At the same time, it also promotes cell apoptosis and worsens GDM [19, 20]. TNF-*α* is mainly distributed on the surface of vascular endothelial cells, which can directly affect islet cells and lead to insulin resistance. Our results showed that the levels of TNF-*α* and IL-6 in the GDM group were significantly higher than those in the control group (*p* < 0.0001). Our study showed that IL-6, TNF-*α*, and IR had a certain correlation, suggesting that TNF-*α* and IL-6 could be used as important indicators for the detection and diagnosis of gestational diabetes mellitus.

ADP is secreted mainly by adipocytes and has 244 amino acids. It has the functions of regulating glycolipid metabolism, acts as an anti-inflammatory, and reduces insulin resistance. Some studies have shown that when GDM occurs, ADP levels decrease significantly and are prefatory to the occurrence of abnormal blood sugar [21]. This study found that the ADP level of the GDM group was significantly lower than that of the control group in newborns, but there were no significant differences in pregnant women with GDM.

Differential expression of TGF-*β*1 seemed to be related with other factors in newborns, but its levels did not differ in the control and GDM-/+ groups of parturient women.

## 5. Conclusions

This study confirms that the downregulation of H_2_S participates in the pathogenesis of GDM and is of great significance in understanding the differences in H_2_S in GDM and normal pregnant women and newborns. The cause of the H_2_S concentration decrease in GDM, how H_2_S participates in the pathogenesis of GDM, and which part of the pathogenesis of GDM is affected by H_2_S are all issues that urgently need to be resolved. Furthermore, the current study suggests that IL-6 and TNF-*α* are correlated with gestational diabetes mellitus, but further study is necessary to explain whether they have causal relationship. In summary, this study expands the research field of H_2_S and provides new insights for exploring further the pathogenesis of GDM.

## Data Availability

The data used to support the findings of this study are included within the supplementary information file(s).

## Supplementary Materials

Supplementary Materials Figure S1: H_2_S concentration did not influence the level of TGF-*β*1 in GDM patients. (A, B) The concentration of H_2_S was negatively correlated with levels of TGF-*β*1 and ADP in pregnant women and newborns. (1C, D) The levels of TGF-*β*1 and glucose were positively correlated in pregnant women but showed no significance in newborns. Figure S2: H_2_S concentration did not influence the level of ADP in GDM patients. (A, B) The concentration of H_2_S was negatively correlated with levels of ADP and ADP in pregnant women and newborns. (C, D) The levels of ADP and glucose were positively correlated in pregnant women but showed no significance in newborns.
