# Supplementary figures and images for: Expression of H2S in Gestational Diabetes Mellitus and Correlation Analysis with Inflammatory Markers IL-6 and TNF-α
Source: J Diabetes Res. 2020 Mar 23;2020:3085840. doi: 10.1155/2020/3085840 (PMC7125483; doi:10.1155/2020/3085840)

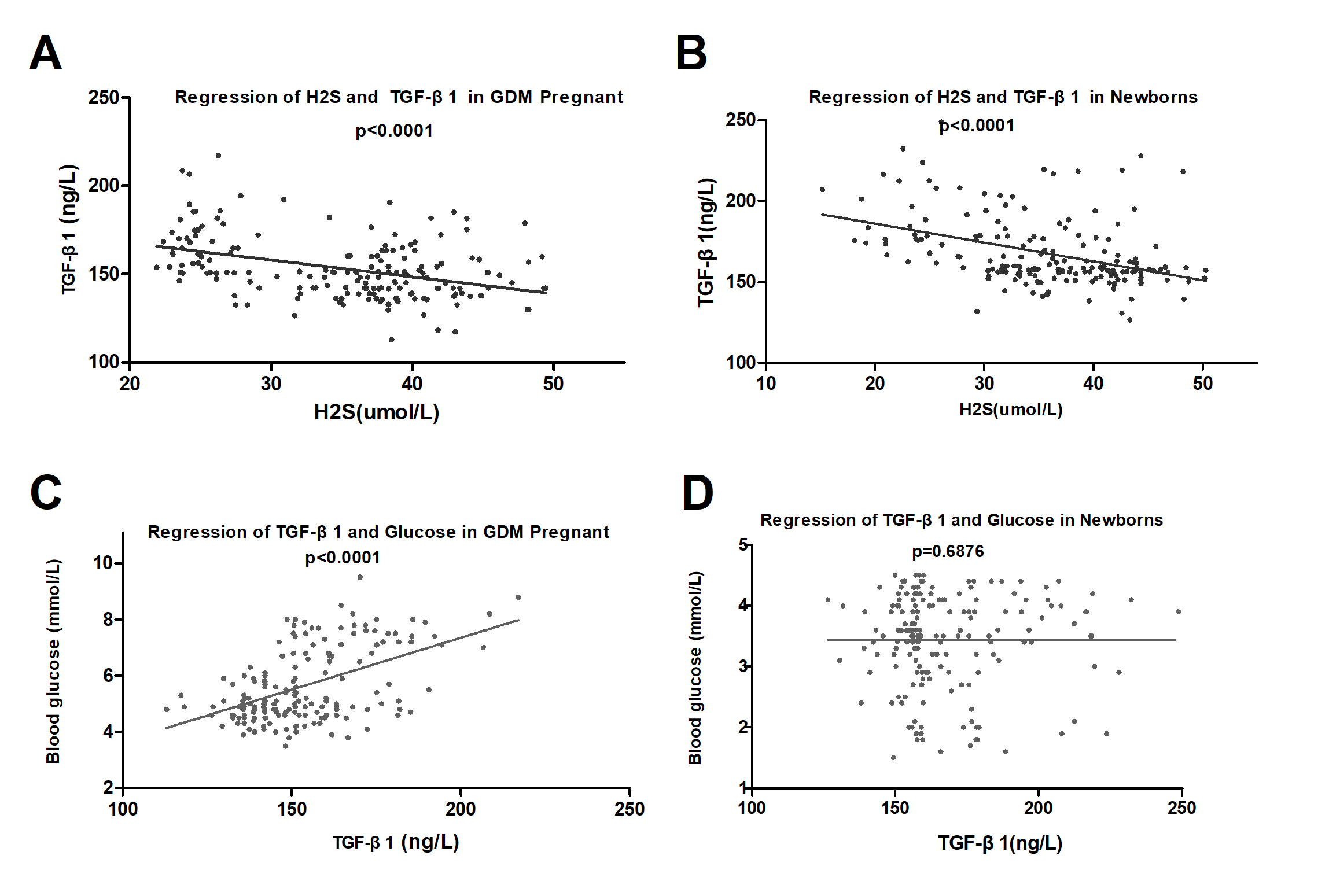

Supplement: Supplementary Materials — Figure S1: H2S concentration did not influence the level of TGF-β1 in GDM patients. (A, B) The concentration of H2S was negatively correlated with levels of TGF-β1 and ADP in pregnant women and newborns. (1C, D) The levels of TGF-β1 and glucose were positively correlated in pregnant women but showed no significance in newborns. Figure S2: H2S concentration did not influence the level of ADP in GDM patients. (A, B) The concentration of H2S was negatively correlated with levels of ADP and ADP in pregnant women and newborns. (C, D) The levels of ADP and glucose were positively correlated in pregnant women but showed no significance in newborns. [file 3085840.f1.zip › FIG S1.jpg]

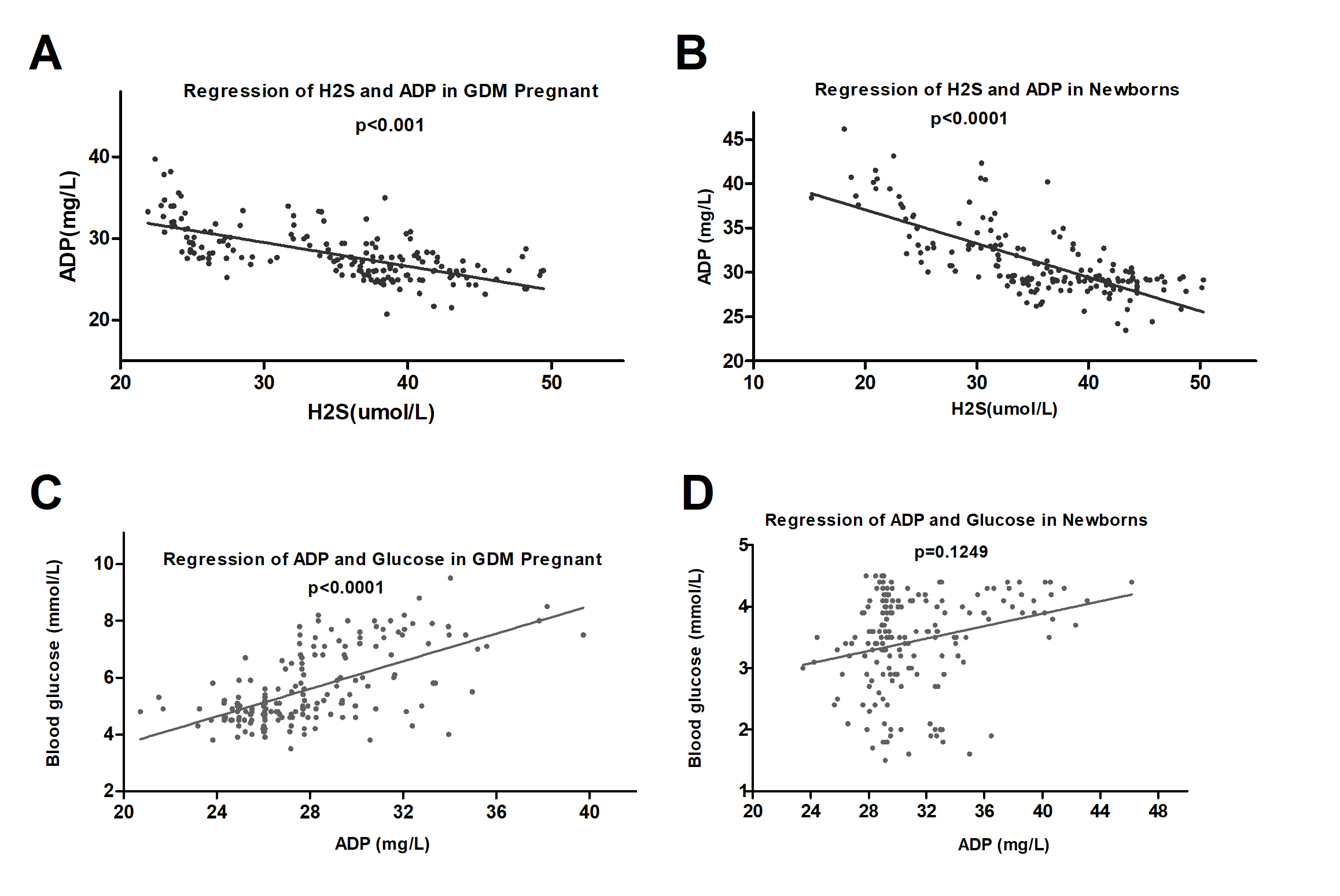

Supplement: Supplementary Materials — Figure S1: H2S concentration did not influence the level of TGF-β1 in GDM patients. (A, B) The concentration of H2S was negatively correlated with levels of TGF-β1 and ADP in pregnant women and newborns. (1C, D) The levels of TGF-β1 and glucose were positively correlated in pregnant women but showed no significance in newborns. Figure S2: H2S concentration did not influence the level of ADP in GDM patients. (A, B) The concentration of H2S was negatively correlated with levels of ADP and ADP in pregnant women and newborns. (C, D) The levels of ADP and glucose were positively correlated in pregnant women but showed no significance in newborns. [file 3085840.f1.zip › FIG S2.jpg]

**A**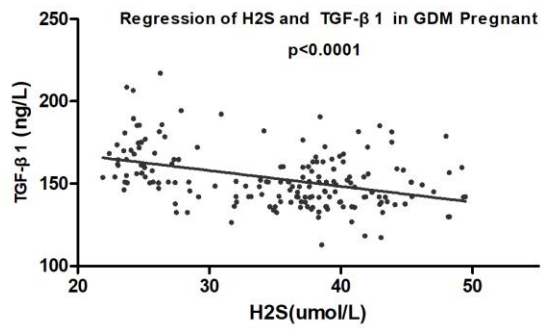**B**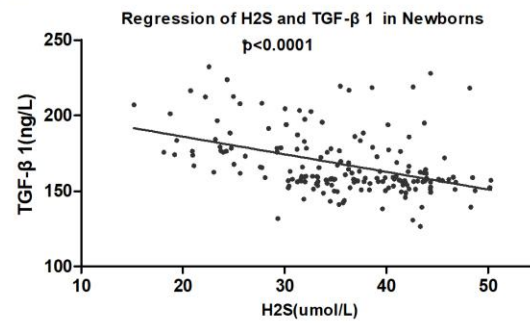**C**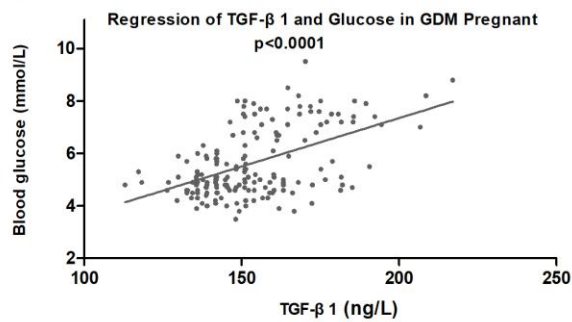**D**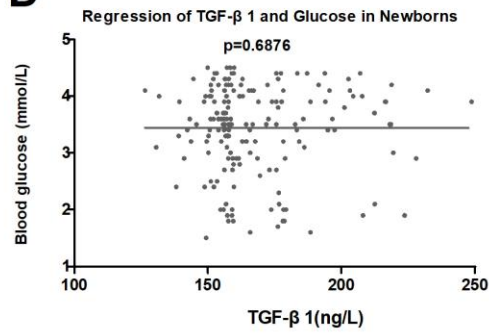**A**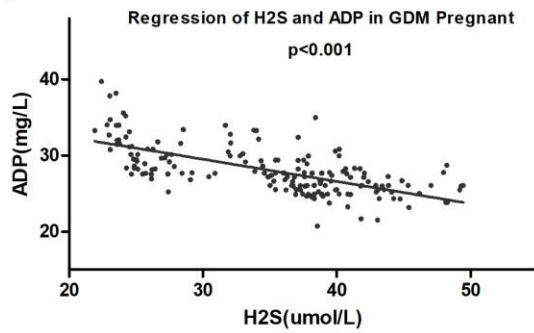**B**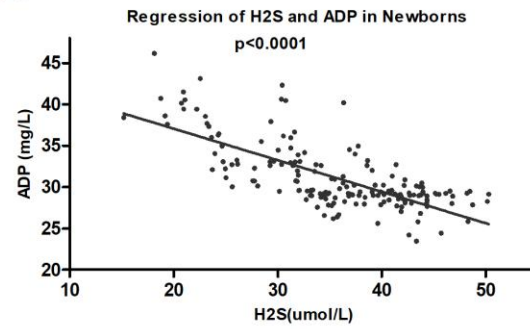**C**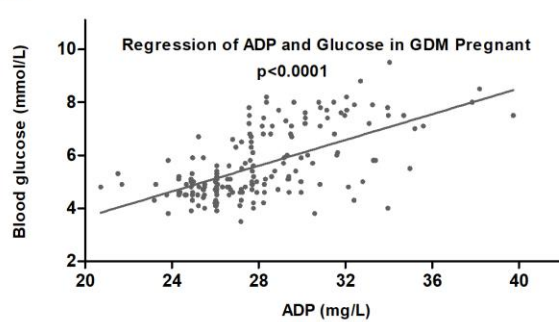**D**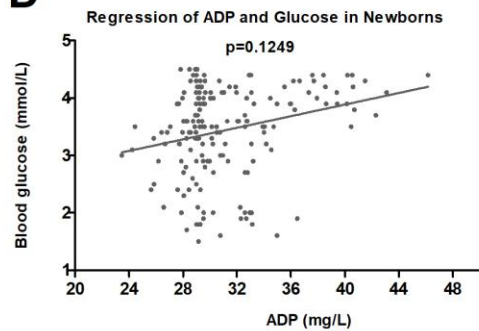

Supplement: Supplementary Materials — Figure S1: H2S concentration did not influence the level of TGF-β1 in GDM patients. (A, B) The concentration of H2S was negatively correlated with levels of TGF-β1 and ADP in pregnant women and newborns. (1C, D) The levels of TGF-β1 and glucose were positively correlated in pregnant women but showed no significance in newborns. Figure S2: H2S concentration did not influence the level of ADP in GDM patients. (A, B) The concentration of H2S was negatively correlated with levels of ADP and ADP in pregnant women and newborns. (C, D) The levels of ADP and glucose were positively correlated in pregnant women but showed no significance in newborns. [file 3085840.f1.zip › mat.3085840.v2.pdf]
